# Antimicrobial Potential of Plastic Films Incorporated with Sage Extract on Chicken Meat

**DOI:** 10.3390/foods10112812

**Published:** 2021-11-16

**Authors:** N. Aziman, M. Jawaid, N. A. Abdul Mutalib, N. L. Yusof, A. H. Nadrah, U. K. Nazatul, V. V. Tverezovskiy, O. A. Tverezovskaya, H. Fouad, R. M. Braganca, P. W. Baker, S. Selbie, A. Ali

**Affiliations:** 1 Alliance of Research & Innovation for Food (ARIF), Faculty of Applied Sciences, Universiti Teknologi MARA, Cawangan Negeri Sembilan, Kampus Kuala Pilah, Kuala Pilah 72000, Negeri Sembilan, Malaysia; ainaziman@uitm.edu.my; 2 Laboratory of Biocomposite Technology, Institute of Tropical Forestry and Forest Products (INTROP), Universiti Putra Malaysia, Serdang 43400, Selangor, Malaysia; 3 Faculty of Food Science and Technology, Universiti Putra Malaysia, Serdang 43400, Selangor, Malaysia; n_azira@upm.edu.my (N.A.A.M.); noorliyana@upm.edu.my (N.L.Y.); nadrahabdhalid@gmail.com (A.H.N.); nazatulumira.karim@gmail.com (U.K.N.); 4 BioComposites Centre, Bangor University, Bangor, Gwynedd LL57 2UW, UK; v.tverezovski@bangor.ac.uk (V.V.T.); o.tverezovskaya@bangor.ac.uk (O.A.T.); r.braganca@bangor.ac.uk (R.M.B.); paul.baker@bangor.ac.uk (P.W.B.); 5 Applied Medical Science Department, Community College, King Saud University, P.O. Box 10219, Riyadh 11433, Saudi Arabia; menhfef@ksu.edu.sa; 6 Scitech Adhesive Systems Ltd., Castle Park Industrial Estate, Flint CH6 5XA, UK; Stewart@scitech-adhesives.co.uk; 7 Centre of Excellence for Postharvest Biotechnology, School of Biosciences, University of Notthingham Malaysia, Jalan Broga 43500, Semenyih, Malaysia; asgar.ali@nottingham.edu.my

**Keywords:** antimicrobial food packaging, *Staphylococcus aureus*, *Escherichia coli*, *Salmonella Typhimurium*

## Abstract

The function of packaging is crucial in the maintenance of fresh meat product quality. This study aimed to assess the efficiency of six films added with coatings 2379L/220 and 2379L/221 (containing sage extracts) to inhibit *Salmonella typhimurium, Staphylococcus aureus*, and *Escherichia coli,* which showed that two of the six films had a significant effect. Additionally, the effects of the films on refrigerated skinless chicken breast meat were evaluated based on microbiological content, colour, weight loss, texture and pH. Four of the six films were examined could extend the storability of refrigerated chicken breast fillets for up to seven days. All six treated films improved the pH, colour stability, weight loss, and texture of the chicken fillets. Therefore, these findings suggested that the coatings containing sage extracts having different viscosities (2379L/220 and 2379L/221) were effective as antimicrobial adhesives in food packaging films and can be commercially applied in prolonging the storage of chicken breast meat without affecting their quality.

## 1. Introduction

The short shelf life of chicken is one of the biggest challenges in the retail industry. [1]. Refrigerated temperatures between 2 and 5 °C are usually used for fresh meat products. Within this temperature range, the growth rate of microorganisms, enzyme activity, and chemical reaction is slowed, thus improving the meat shelf life [2,3]. However, refrigerated meat is still perishable due to metabolic changes occurring during storage and microbial spoilage that result in physiological ageing and deterioration in the colour, texture, and flavour. The propensity of fresh meat products to microbial contamination and spoilage is increased due to its high water content, leading to a short shelf life (3–5 days) [4]. Refrigerated chicken meats showed a higher risk (*p* < 0.05) of *Salmonella* contamination during storage compared with other stages from chicken processing to retail [5]. In response, food industries have urged the need for innovations in food packaging as a strategy to maintain a low microbial content and high food quality to extend the shelf life of poultry products. This extended shelf life may be achieved by using an active packaging approach.

Active packaging is regarded as a smart packaging method by packaging with active ingredients in which a few types of additives/active materials are included within the packaging film or are located at the surface of the packaging containers [6]. It performs an additional function beyond the basic safety functions of traditional packaging methods [7] and comprises interactions among the food products, active substances, and inner and outer space conditions of the packaging materials. The principle of active packaging is based either on the addition of certain ingredients used in a polymer or particular polymer properties used as the packaging material [8]. The use of active food packaging minimizes hazards and improves the quality and safety of foodstuffs [6]. The utilisation of active packaging to increase the shelf life and quality of meat products has been well-reported in several studies. In particular, the use of rosemary and oregano in an active film for lamb steaks improved their oxidative stability, and the colour was maintained up to 8–13 days of storage [9]. The shelf life of poultry meat products contained within modified atmospheric packaging at 1 ± 1 °C were extended up to two-fold [10]. The storability of refrigerated chicken was extended to 14 days by treatment with modified atmosphere packaging method added with a nisin–ethylenediaminetetraacetate (EDTA) antimicrobial agent [11]. From both microbiological and sensorial analyses, the half-cooked laminated chicken meat prolonged its shelf life up to 7 to 8 days at 4 °C by the treatment of EDTA–lysozyme solution added with rosemary oil and oregano oil via vacuum packaging [12]. The incorporation of chitosan or chitosan–oregano in modified atmosphere packaging showed no influence in the taste of chicken breasts stored at refrigerated storage period (4 °C) and were acceptable after 21 days [1]. Chitosan coating (2%) lowered of the population of *Staphylococcus* spp. on ready-to-cook chicken, thus extending its shelf life [13].

The leaves of *Salvia officinalis* L. (sage) extracts were examined for their antimicrobial activities. The aqueous extract of sage exhibited significant antibacterial activity against *Bacillus mycoides, Proteus* sp., *Bacillus subtilis*, and *Enterobacter cloacae,* while *Escherichia coli* was the most resistant [14]. The 96% ethanol extract of sage showed a strong antimicrobial effect against *Bacillus subtilis, Staphylococcus aureus*, and *Aspergillus niger*, with MIC values of 6, 10, and 30 µL/mL, respectively [15]. The hydro-ethanol extract of sage significantly reduced the bacterial proliferation in fish fillets [16]. Oleanolic and ursolic acids were detected in a 70% acetone extract of sage, and these components also exhibited an antibacterial effect on methicillin-resistant *Staphylococcus aureus* [17]. The major polyphenolic compounds identified in the 70% ethanol extracts of sage were caffeic acid, coumaric acid, rosmarinic acid, quercetin, and myricetin [18]. In addition, the essential oil of sage also exhibited antimicrobial activity. The sage essential oil (20 μL/mL) showed antibacterial activities against *Staphylococcus aureus* [19]. The sage essential oil also exhibited high antibacterial activity against *Candida albicans* and *Staphylococcus aureus*, while its major components were borneol (8.33%), α-thujone (41.48%), β-thujone (6.75%), camphene (3.46%), virdiflorol (5.85%), α-pinene (3.24%), β-pinene (2.25%), α-humulene (2.64%), and 1,8 cineole (7.94%) [20]. All these extracts provided good antimicrobial properties; however, the phenophase in which the collected leaf could influence their biological activity [21]. 

Antimicrobial packaging is the addition of antimicrobial agents into packaging systems with the objective to enhance the lifespan of food products while preserving its safety and quality. To date, the application of sage extracts in antimicrobial packaging is limited. Most recently, poly(ε-caprolactone) (PCL) film incorporated with sage extract (solid dispersion) was produced and studied [22]. They showed an antibacterial effect against *Staphylococcus aureus* and *Escherichia coli*, which revealed a remarkable potential for applications of a sage extract in antimicrobial food packaging. The *Aspergillus flavus* growth on pistachio kernels was totally inhibited by whey protein concentrate-based coating incorporated with 4000 ppm of sage extracts [23]. Therefore, the utilisation of sage extract as an antimicrobial agent in packaging film was conducted in this study. In addition, with a surface coating of commercial grades of plastic films employed in the present study, coupled with significantly lower loads of the sage extract, might represent a better economical solution.

The aims of the present work were (1) to study the effectiveness of plastic films incorporated with two antimicrobial adhesives 2379L/220 and 2379L/221 (containing sage extracts) against *Salmonella typhimurium, Escherichia coli,* and *Staphylococcus aureus* and (2) to examine the efficacy of coatings on the chemi–physical properties of refrigerated chicken breast meats.

## 2. Materials and Methods

### 2.1. Materials and Chemicals

Dried crushed plant materials of food grades, namely sage (*Salvia officinalis* L.), were purchased from SLP Foods (UK). Two grades of coatings (adhesives) containing sage extracts, namely 2379L/220 and 2379L/221, were supplied by Scitech Adhesive Systems Ltd. (UK). In this study, 2379L/220 and 2379L/221 were coated onto different films of different thicknesses. Four grades of food packaging films were supplied by Parkside Flexibles Ltd. (UK): 12-µm PET, 35-µm Biopolymer TP302 (originally from TIPA), 23-µm NatureFlexTM NVR (originally from Futamura), and 52-µm HCFD2 compostable laminate. 

### 2.2. Preparation of Extracts

Sage from the Mediterranean region was extracted using ethanol as reported in a previous study [24]. In this study, however, for the production of strong and uniform coatings on a surface of polymeric films, 96% ethanol was used, as these extracts provided improved compatibility with other components in the coating formulations. A total of 600 g of the dried sage plant material was blended in 2 L of ethanol using a laboratory blender at room temperature to reduce the particle sizes. The sage blend in ethanol was transferred into a 10-L reactor flask, equipped with a mechanical stirrer, air condenser, and N_2_ inlet. A further 4 L of ethanol was added, the suspension stirred under a nitrogen blanket (to avoid oxidation of polyphenols) and refluxed at 78 °C for 30 min. Evaporation of the solvent under reduced pressure resulted in a sage extract. The extract was freeze-dried to a constant weight. The average yield of the total soluble solids (calculated per weight of dried material) was 100.7 g (16.8%).

### 2.3. Preparation of Coated Films

Two grades of coatings (containing sage extracts, namely 2379L/220 and 2379L/221) were used in the preparation of coated films. Both grades were heat-sealable coatings designed for food packaging applications and based on aqueous compostable polyurethane (PU) dispersions, which were formulated by Scitech Adhesive Systems Ltd. (Table 1). In this method, the stability of the PU is important. The PU are based on aqueous dispersions to reduce the environmental concerns associated with organic solvents used in PU synthesis during processing and application of the polymer. 

The 6 g/m^2^ wet of the 2379L/220 and 2379L/221 coatings were coated on four grades of food packaging films (12-µm PET, 35-µm Biopolymer TP302, 52-µm HCFD2 compostable laminate, and 23-µm Natureflex NVR). The targeted antimicrobial film thickness was 1.9 µm, with the sage extract dispersed at 0.15 g/m^2^. Six formulations of films were analysed as shown in Table 2.

### 2.4. Determination of Antimicrobial Activity of Coated Films

For the antimicrobial analysis, two samples were prepared from each film. Both samples were labelled with numbers: 1 or 2. The antibacterial activities of the six coated films against *Staphylococcus aureus, Escherichia coli*, and *Salmonella typhimurium* were determined according to the ISO 22196 method [25] with some changes. This assay was repeated in triplicates.

#### 2.4.1. Microbial Cultures Preparation

*Salmonella typhimurium* (ATCC 14028), *Escherichia coli* (ATCC 11229), and *Staphylococcus aureus* (ATCC 6538P) strains (Microbiologics, KWIK-STIKTM 2 pack, Saint Cloud, MN, USA) were bought from Choice-Care Sdn. Bhd, Kuala Lumpur, Malaysia. The bacteria cultures were incubated for 24 h in an incubator shaker (200 rpm) at 30 °C. The active cultures were diluted with 1/50 diluted LB broth, and their optical density (OD) was adjusted into 1 to 2 × 10^5^ CFU/mL (known as the start). 

#### 2.4.2. Modified ISO 22196 Method

Approximately 0.4 mL of microbial broth was transferred on each film (5 cm × 5 cm), and then, another piece of same film (4 cm × 4 cm) covered the top of the microbial broth. The plates were incubated for 24 h at 30 °C. About 4.6 mL of 0.85% NaCl was added to each sample. Serial dilution was conducted with 100 μL of each dilution (dilution 10^−1^ until 10^−6^) were spread plated into LB agar plates, then incubated for 24 h at 30 °C. This step was repeated in triplicate. The positive and negative controls used were gentamicin (G) and no film (no sample), respectively. The volume of gentamicin used was 100 µL of 1 µg/mL. The results were expressed as the log CFU/mL sample. The actual number in a culture dish containing 30–300 colonies was calculated based on Equation (1):

Total count (log CFU/mL sample) = log [C∗D/V](1)

where C: number of colonies in the selected dilution, D: dilution factor, and V: volume of diluted sample used (mL).

### 2.5. Shelf Life

The shelf life examination, including the microbiological activity and quality assessment, was determined on days 0, 1, 3, 5, and 7 of storage. As for the control, a polypropylene (PP) microwavable container (700 mL) was used. However, for the treated samples, the films were manually laminated onto a kraft paper tray (11.5 × 9 × 5 cm) aseptically. 

#### 2.5.1. Determination of Microbiological Activity of Coated Films

##### Sampling and Storage

Fresh chicken breast fillets were bought from a supply chain company, Segi Fresh (Balakong, Selangor, Malaysia). The chicken breast meat samples weighing approximately 30 ± 3 g were placed on the laminated food tray and covered with the same lamination film. Then, the trays were kept for 7 days at 4 °C in refrigerator [26]. The standard total plate count (TPC) was evaluated according to the CLSI method [27]. The chicken meats used for each treatment were carried out in triplicate during storage. TPC on the initial day (day 0) was conducted upon freshly bought chicken breast meat samples with no treatment applied. 

##### Total Plate Count (TPC)

Approximately, a 25-g meat sample from a chicken breast was added to 225 mL of a 0.1% sterile peptone water solution within a stomacher bag, then homogenised by a stomacher (Tekma Lab Blender 80, Seward Medical, Worthing, West Sussex, UK) for 60 s. Serial dilutions were prepared for each sample; then, the homogenates (0.1 mL) were spread on Plate Count Agar (PCA, Oxoid, UK). Those inoculated plates were then placed in an incubator for 18–24 h at 37 °C. TPC was expressed as the log CFU/g [28].

#### 2.5.2. Quality Assessment

##### Colour Analysis

The pictures of the chicken breast meat samples were taken with a camera (Apple iPhone 6 Plus, Apple Inc., Cupertino, California, United States), and the colours of the samples were observed throughout the storage. The colours with respect to a*: redness, b*: yellowness, and L*: lightness for the chicken breasts were determined using a chroma meter (CR-410, Konica Minolta, Japan). The measurements were made at three different locations per sample [29]. To obtain an average value, the mean values of L*, a*, and b* of each sample were calculated from triplicate chicken samples [30].

##### Weight Change Analysis

The weight changes of the chicken samples were determined from the beginning to the end of storage. The weight change of the samples was calculated [28] using Equation (2):

Weight change (%) = (W_1_ − W_2_) / W_1_ × 100 (2)

where W_1_: initial weight of sample, and W_2_: weight of sample during storage.

##### Texture Analysis

The textures of the chicken breasts were analysed using a texture analyser (TA.HDplusC, Stable Micro Systems Ltd., Godalming, UK). The textures were described in terms of firmness, where measurements were made at three different locations per sample. Mean values of the triplicate chicken samples were analysed to determine the average value of the hardness (N) [31]. 

##### pH Analysis

The pH changes of the chicken breast meats were determined following the AOAC method [32]. Approximately, 10 g of chicken meat underwent homogenisation with 100 mL of distilled water, and the pH measurements were performed after filtration using a pH meter (Mettler Toledo, Greifensee, Switzerland USA) to examine the pH changes of the filtrates.

### 2.6. Statistical Analysis 

All results were performed for the statistical analyses by applying a software of Statistical Analysis System (SAS) (SAS Institute, Vr. 9.1.3, Cary, NC, USA). Data in triplicate were analysed using an ANOVA analysis and expressed as the mean ± standard deviation values. 

## 3. Results and Discussion

### 3.1. Antimicrobial Activity of Coated Films

**Figure 1:**
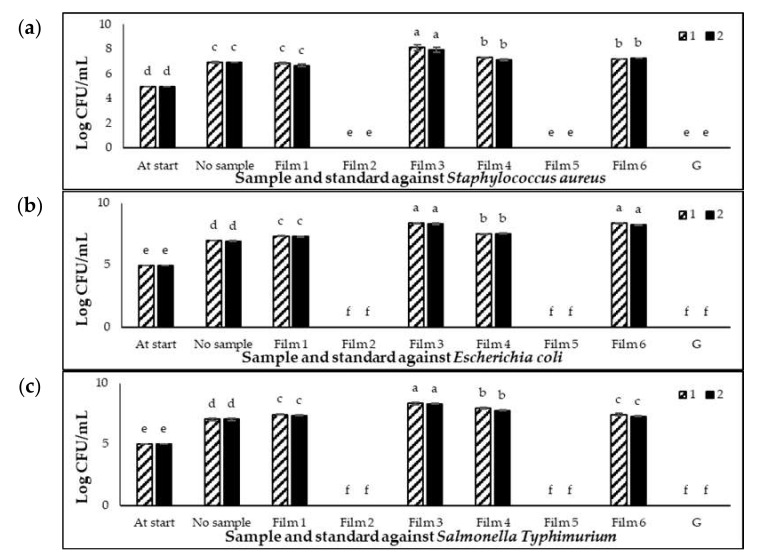
(**a**) Growth of *Staphylococcus aureus*, (**b**) growth of *Escherichia coli,* and (**c**) growth of *Salmonella Typhimurium* on films. At the start: 5 log CFU/mL of the culture cell number before incubation. 1: Location 1, 2: Location 2, No sample: no film, and G: Gentamicin. Error bars indicate the standard deviation (*n* = 3). Small letters of a–f are different significantly (*p* < 0.05) amongst the samples.

Figure 1a–c showed antimicrobial activities of the six films, gentamicin (positive control), and no sample (negative control) against *S. typhimurium*, *E. coli*, and *S. aureus*, respectively. A log10 value of 5 was standardised as the start value for each culture with the same cell number. Based on the figures, all three bacteria populations increased (*p* < 0.05) from the start of the experiment to a log10 value of 7 for no sample treatment.

In comparison to the no sample treatment (Figure 1a), Films 3, 4, and 6 showed the highest population of *S. aureus*, and Film 1 exhibited no difference (*p* > 0.05) with the no sample treatment (negative control). The slightly higher counts associated with some of the films might be due to the higher rate of oxygen transfer occurring through the thin liquid cell suspensions, whereas the oxygen transfer within the larger liquid volume associated with the negative control may be slightly impaired. However, the *S. aureus* population was totally reduced (*p* < 0.05) by Films 2 and 5, and they were comparable with the positive control, gentamicin. Based on Figure 1b,c, the activity of all films inhibited *E. coli* and *S. typhimurium*, also showing similar trends with the film samples against *S. aureus*, where Films 1, 3, 4, and 6 showed the highest population compared to the no sample treatment, while Films 2 and 5 exhibited a total reduction (*p* < 0.05) of the *S. typhimurium* and *E. coli* populations. Overall, Films 2 and 5 showed the highest bactericidal effect on *S. aureus*, *E. coli*, and *S. typhimurium*. We found that the incorporation of adhesives with sage extracts 2379L/220 and 2379L/221 at 6 g/m^2^ wet on 35-µm biopolymer TP302 showed strong preservative effects compared to the others. It was assumed that the underlining plastic films onto which the sage extracts were coated would not exert any influence in the antimicrobial properties being physically separated from the microorganisms in suspension around the chicken breasts by the coated layer. Furthermore, there was no previous studies on the antimicrobial effects of these films. However, it appeared that the biopolymer TP302 did have an effect. Previous research has shown that the presence of a natural stabiliser, rosin derived from pine needles that itself has an antibacterial effect, complemented the antibacterial activity of the coated chitosan layer [33]. This is one possibility, but other factors need to be considered. For instance, the antimicrobials could effectively penetrate the 35-µm biopolymer TP302 film compared to other plastic films.

Even though there is scarcity of data on the efficacy of sage extracts in packaging films, the effectiveness of sage extracts as an antimicrobial agent has been well-reported by previous studies. The use of an 80% ethanol extract of sage exhibited antibacterial activity on *P. aeruginosa* and *S. aureus* [34]. A methanol extract of sage at 500 mg/mL showed the highest antimicrobial activity compared with hot and cold aqueous extracts against *Staphylococcus aureus, Escherichia coli*, and *Pseudomonas aeruginosa* with plate hole diffusion diameters of 30 mm, 28 mm, and 20 mm, respectively [35]. The bioactive compounds of sage extracts may have an important role in protecting plants from microbial attacks. These compounds are mainly phenolics, terpenoids, polyphenols, and flavonoids [36]. The mechanisms of these compounds against microorganisms vary and are not clearly known; however, some research has been carried out in this area. By inhibiting the function of the cytoplasmic membrane, flavonoids would also inhibit DNA gyrase and β-hydroxyacyl-acyl carrier protein dehydratase [37,38]. The potential mechanism of the antimicrobial activity of sage extracts is their capability to destroy the cellular membranes of bacteria [39]. As reported by Aljuraifani [40], the mechanism of antimicrobial action may involve enzymatic inhibition by oxidised compounds, serving as a source of stable-free radicals that inactivated proteins and caused its loss of function. However, antimicrobial action would only occur due to a mixture of chemicals interacting with an additive or in a synergistic way rather than a single molecule.

Several previous studies reported the effectiveness of antimicrobial agents in films. The maximum zone size (1.157 mm^2^) against *E. coli* was found with the combination of antimicrobial protein lysozyme (66 mg/g of film) and 30 mM of EDTA in a cast corn zein film [41]. The bacterial growth was inhibited by the pea starch films containing grape seed extracts to a log10 value of 1.3 after four incubation days at 4 °C [42]. Chitosan biopolymer films exhibited an antimicrobial activity against *S. typhimurium* and *E. coli* 0157:H7 at the concentration of 104 CFU/mL [43]. Chitosan films showed an inhibition effect against yeasts, bacteria, and fungi with values of 0.47–2.96 log10 reductions, while chitosan films with a 5% ethyl-Nα-dodecanoyl-l-arginate antimicrobial compound exhibited antimicrobial activity with the values of 1.78–5.81 log10 reductions [44].

### 3.2. Shelf Life Study

#### 3.2.1. Total Plate Count (TPC)

**Figure 2:**
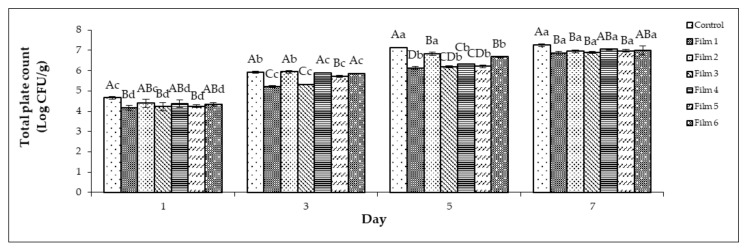
The total plate counts (TPC) of chicken meat stored in the PP containers and plastic film laminated trays during seven storage days at 4 °C. Control: polypropylene (PP) microwavable container. Error bars indicate the standard deviation (*n* = 3). Capital letters of **A**–**D** are different significantly (*p* < 0.05) for each day amongst the treatments. Small letters of **a**–**d** are different significantly (*p* < 0.05) for each sample amongst the storage days.

The total plate counts (TPC) for chicken breast meat stored in a PP container (control) and plastic film laminated trays during seven storage days at 4 °C are shown in Figure 2. The values increased in all treatments up to seven days with log10 values between 4.17 and 7.26. It is most likely that the majority of the bacterial growth occurred at the surface of the chicken meat, because the rates of the oxygen transfer would be higher.

The initial TPC of the chicken breast meat without any treatment (day 0) was a log10 value of 4.13, and this was similar to the previous study [45] at a log10 value of 4.85. The limit of acceptability for fresh edible chicken meat is 7.00 log CFU/g [46]. Refrigerated chicken meat revealed a TPC exceeding 8 log CFU/g, with a pH higher than 7 when stored for more than seven days [47]. In this work, chicken meat stored in a controlled container showed a rapid increase in bacterial population that exceeded the limit of acceptability from day 5. However, among the treated films, only the chicken breast meat stored in Films 4 and 6 exceeded the limit of acceptability, although these values were significantly lower (*p* < 0.05) compared with the control on day 7. Meanwhile, the chicken breast meat stored in Films 1, 2, 3, and 5 showed the lowest TPC values and were still acceptable up to seven days, although these chicken meats showed only a minor difference in bacterial populations, yet were significant different (*p* > 0.05) compared with Films 4 and 6 that showed a slightly higher bacterial growth. Thus, this indicates that Films 1, 2, 3, and 5 exhibited the highest antimicrobial properties compared with the other films. The TPC results in this work were supported by the antimicrobial analysis of the coated films (Section 3.1), especially Film 2 and Film 5, which exhibited a killing effect against *S. typhimurium*, *E. coli*, and *S. aureus*, thus showing a preservative effect on the stored chicken samples in the refrigerator. However, Films 1 and 3, which showed good TPC results, were ineffective against *S. typhimurium*, *E. coli*, and *S. aureus*, as discussed in Section 3.1. 

A previous study showed that the total aerobic mesophilic bacteria count on chicken thigh meats surpassed the limit of acceptance at the 7th day for the control sample without modified atmosphere packaging but surpassed this limit at day 19 for the sample with modified atmosphere packaging and iron-based oxygen scavengers [48]. Another study showed the extended lifespan of chicken wrapped with a chitosan film, and 2% *Trachyspermum ammi* essential oil had an antimicrobial activity (*p* < 0.05) on the total psychrophilic, total aerobic, and coliform bacteria [49]. However, the advantage of using sage is its ease of accessibility as a plant product. A polyethylene nanocomposite film filled with silver nanoparticles prepared using modified atmospheric packaging method could extend the chicken breast fillets shelf life [50].

Hence, the application of antimicrobial packaging with sage extract in this study showed an improvement in the chicken breast meat storability. The efficiency of sage extract as an antimicrobial agent in food packaging film is associated with its transmittance rate from films onto the surfaces of foods. This diffusion rate of antimicrobial agents can be affected by several factors. As reported by Ripoche [51], the diffusion of nisin (natural antimicrobial agent) can be affected by the types of films used (edible or biopolymer), nisin concentration, and storage conditions. The success of an antimicrobial film primarily relies on the selection of the antimicrobial agent, where antimicrobials are chosen by taking into account the type of food packed and, also, the associated deteriorative microorganisms [52]. The well-known antimicrobials like natural extracts, enzymes, organic acids, bacteriocins, chelators, and metal ions that are usually incorporated into biobased and functionalized synthetic polymers showed excellent controlled release behaviour when in direct contact with the food surfaces [53].

#### 3.2.2. Colour Changes

**Figure 3:**
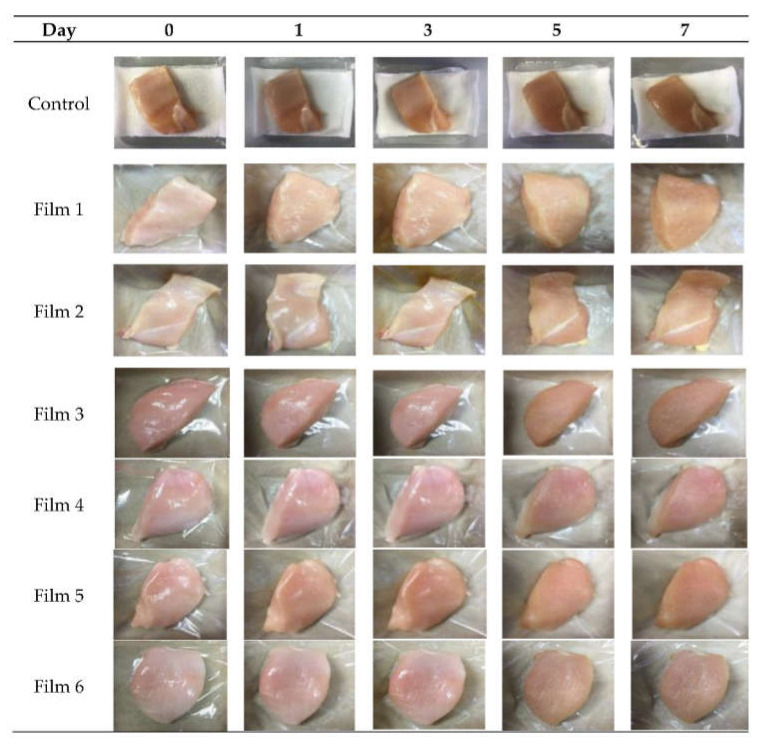
Overall visual quality of chicken breast meats stored in a PP container (control) and plastic film laminated trays during seven storage days at 4 °C.

Colour is an essential factor that affects consumers’ liking for poultry meat [54]. The observations of the chicken breast meats stored in PP containers (control) and plastic film laminated trays during seven storage days at 4 °C are shown in Figure 3. 

**Figure 4:**
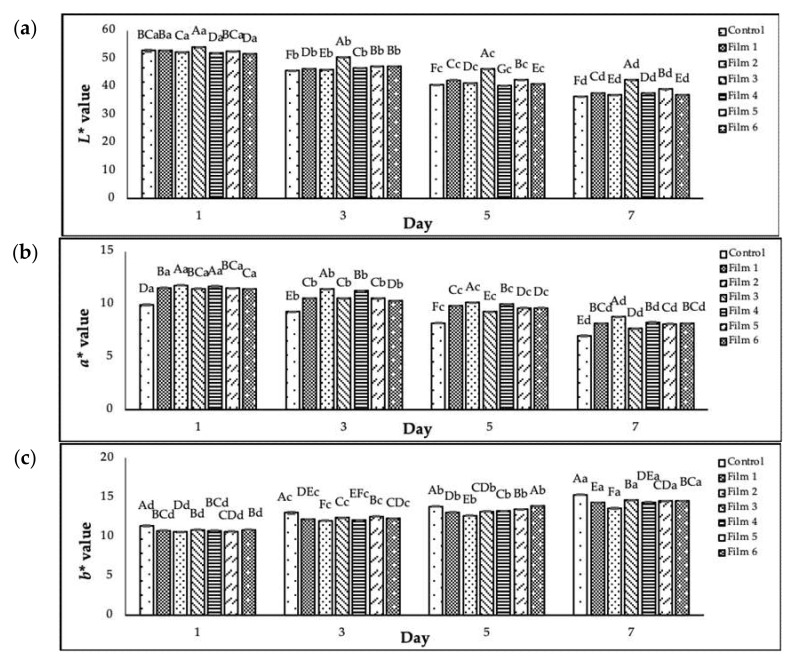
(**a**) The values of L*, (**b**) the values of a*, and (**c**) the values of b* for chicken meat stored in a PP container and plastic film laminated trays during seven storage days at 4 °C. Colour parameters: L*, a*, and b*. Control: polypropylene (PP) microwavable container. Error bars indicate the standard deviation (*n* = 3). Capital letters of **A**–**F** are different significantly (*p* < 0.05) for each day amongst the treatments. Small letters of **a**–**d** are different significantly (*p* < 0.05) for each sample amongst the storage days.

According to Figure 3, chicken breast meats in the PP container without any antimicrobial coating turned the darkest compared to the other samples. In this study, the initial values of L*, a*, and b* for the chicken meat without any treatment (day 0) were 56.02 ± 0.21, 12.01 ± 0.08, and 10.29 ± 0.12, respectively. The colours (L*, a*, and b*) for the chicken meats stored in the PP container and plastic film laminated trays during seven storage days at 4 °C are presented in Figure 4.

Based on Figure 4, the values of L* and a* for all chicken meats decreased (*p* < 0.05) gradually over seven days of storage, and these were greater for the control sample. The L* values were between 36.50 and 54.11, and the a* values were between 7.00 and 11.78. The decrease of L* and a* values for all chicken breast meats where the chicken breast meats had darkened during the seven days of the storage may be caused by protein decomposition within the muscle [55]. Besides, the darkening of the chicken meats may be due to the reduction of the oxygen level at the surface tissue caused by microbial growth [47]. This oxygen level reduction promotes the oxidation or denaturation of myoglobin and the formation of deoxymyoglobin, resulting in the degradation of the red colour within chicken meat. However, the chicken breast meats showed increased (*p* < 0.05) b* values over the storage period. The changes in meat pigmentation during storage [56] was evident by the increase in the b* values due to the progression of meat spoilage primarily occurring through oxidative processes [57].

In general, the redness and lightness of chicken meats might have affected by the type of container. After the 7 days storage period, the values of L* and a* for the treated chicken breast meats stored in laminated plastic film trays were significantly greater (*p* < 0.05) than those stored in the PP containers (control), indicating that the red colour within these treated chicken breast meats was more intense than the colour of the control sample. The chicken breast meat stored in Film 3 showed the highest (*p* < 0.05) L* value (42.36 ± 0.06), followed by Film 5 > Film 1 > Film 4 > Film 2 = Film 6 > control. However, Film 2 showed the highest (*p* < 0.05) a* value (8.81 ± 0.04), followed by Film 4 ≥ Film 1 = Film 6 ≥ Film 5 > Film 3 > control. These results indicate that the colour of chicken meat stored in the treated plastic films with sage extracts was maintained throughout the 7 days of storage period in this study.

The incorporation of a natural antimicrobial agent, chitosan, to the chicken breast meat packed with a modified atmosphere exhibited greater L* values (*p* < 0.05) compared with the control sample [1]. The addition of a natural antimicrobial agent, marjoram essential oil, in dipping treatments (based nano-emulsion technique) to raw chicken drumsticks significantly reduced the colour loss of the meat during refrigerated storage for 12 days [58]. Additionally, moisture loss contributed to the colour changes in refrigerated chicken breast meat, where the higher the moisture loss, the darker the chicken meat [54]. 

#### 3.2.3. Weight Loss Changes

The weight loss changes of the chicken breast meats stored in PP containers (control) and laminated plastic film trays during seven storage days at 4 °C are summarized in Table 3. The weight loss of the samples increased (*p* < 0.05) after seven days of storage for the control samples, while the treated films ranged between 3.16 and 6.83%. 

The control samples and the treated samples were significantly different (*p* < 0.05) on the first day storage, where the control samples had a higher weight loss. Following the storage, the chicken meats sealed in Film 2 showed the lowest (*p* < 0.05) weight loss (6.19 ± 0.04%), followed by Film 1 = Film 6 < Film 3 = Film 5 < Film 4 < control. The observed mass loss can be mostly attributed to the moisture loss with minor proportions of loss associated with enzymatic reaction and the microbial activity, causing the acidification of the surface of chicken meat, thus influencing the weight loss changes. The rate of moisture loss from the chicken meat is high, while its water-holding capacity is reduced gradually during storage, resulting in shrunken meats with a significant weight loss [59,60]. Besides, microbiological growth is another factor that influences the ability of myofibrils water retention in meat during storage in cold conditions [61,62]. This result is also correlated with the TPC value of all the samples in Section 3.2.1, where the highest TPC value of the control samples contributed to its high weight loss. Furthermore, the high weight loss of the control samples in this study contributed to the darkest colour of chicken breast meats stored in PP containers (control), and these results supported the colour results described in Section 3.2.2. Overall, the treated plastic films controlled the weight loss of chicken breast meat in this study. The deterioration of the chicken breast fillets coated with edible coatings of isolated soy protein and guar gum and incorporated with antimicrobial agents nisin and oregano oil occurred more slowly with low weight loss and mild changes in the pH and colour during storage under refrigerated conditions compared to the uncoated samples [4].

#### 3.2.4. Hardness Changes

The texture analysis of the chicken breast meats stored in PP containers (control) and plastic film laminated trays during seven storage days at 4 °C was recorded as the hardness, as tabulated in Table 3. In this study, the initial value of hardness for the chicken meats without any treatment (day 0) was 117.21 ± 0.13 N. The results showed that the chicken breast meat undergoes a hardening process, as revealed by the gradually increasing hardness values (*p* < 0.05) during storage, with values between 118.80 and 144.02 N. Meat tenderness is due to the proteolysis of key myofibrillar proteins, thus preserving the structural integrity in muscle fibres [63]. 

The chicken breast meat stored in Film 2 also showed the lowest (*p* < 0.05) hardness value (137.69 ± 10.23 N), followed by Film 1 = Film 6 < Film 3 < Film 5 = Film 4 < control after the storage period. This similar trend was also observed for the weight loss of the chicken meat (Section 3.2.4). The increase in hardness on day 7 of the refrigerated chicken breast meat was associated with a high moisture loss. The moisture loss from the chicken meat during storage resulted in a weight loss and subsequent hardening of the meat texture [64]. A higher weight loss of the chicken meats kept in PP containers (control) compared to the chicken meats in the treated films led to an increase in hardness, indicating that the treated plastic films could control the moisture loss and texture of the chicken breast meat.

#### 3.2.5. pH Changes

The pH changes of chicken breast meat stored in PP containers (control) and laminated plastic film trays during seven days of storage at 4 °C is shown in Table 3. The initial pH value of the chicken samples without any treatment (day 0) was 6.15 ± 0.06. The pH values increased (*p* < 0.05) gradually during seven days of storage period for all control samples, while chicken meats in the treated films ranged between pH 6.20 and 7.13. Previous research has shown the pH change, formation of slime, degradation, production of odours and changes in physical appearance could be attributed to the microbial spoilage [65].

The pH of the chicken breast meat stored in treated films exhibited lower values (*p* < 0.05) than the control samples from day 5 until day 7. After storage, the chicken meats stored in Films 1, 2, and 5 exhibited the lowest pH values (*p* < 0.05) among all treated films, followed by Films 3, 4, and 6 that were similar to one another. However, the chicken breast meats stored in PP containers (control) showed the highest pH value (7.13 ± 0.67), where the pH value above 7 is considered as having negative sensory attributes [64]. The pH incremental increases during storage might be caused by the utilisation of amino acids, which are released during protein degradation and after depletion of stored glucose by bacteria [62]. In addition, the pH changes might be promoted by the production of acids and basic nitrogenous substances (ammonia and amines) from the spoilage microorganisms [47,66,67]. In this study, the treated plastic films significantly inhibited incremental pH increases (*p* < 0.05) compared to the control from day 5 onwards during storage at 4 °C. Evidence is corroborated by the highest TPC values of the chicken breast meats obtained when stored in PP containers (control), and these control samples also exceeded the limit of acceptability from day 5 as compared to the treated samples. Therefore, it is highly likely that the bacterial population associated with the control samples may cause the accumulation of bacterial by-products such as derived from amino acid decomposition, thereby leading to increasing pH.

Treatment of raw chicken meat with rosemary and clove caused a lowering of the pH to 5.48 ± 0.06 compared to the control, 6.66 ± 0.02 [62]. They also reported that the lower pH of the treated samples was associated with the inhibitory effect of antimicrobial compounds found in rosemary and clove extracts, suppressing the cellular division of spoilage microorganisms, which metabolise basic nitrogen compounds. Moreover, the change in pH has also been linked to the colour variation of chicken breast meat [60].

## 4. Conclusions

The results prove the effectiveness of antimicrobial packaging in inhibiting bacterial growth, while improving colour, weight loss, texture, and pH, thus prolonging the shelf life of chicken breast meat during seven days of storage at 4 °C. Films 2 and 5 (incorporation of 2379L/220 and 2379L/221 at 6 g/m^2^ wet on 35-µm biopolymer TP302) showed a bactericidal effect on *S. aureus*, *E. coli,* and *S. typhimurium*. However, the strongest preservative effect on chicken breast meat quality was achieved by incorporation of 2379L/220 coated at 6 g/m^2^ wet on 12-µm PET (Film 1), 2379L/220 coated at 6 g/m^2^ wet on 35-µm biopolymer TP302 (Film 2), 2379L/221 coated at 6 g/m^2^ wet on 52-µm HCFD2 compostable laminate (Film 3), and 2379L/221 coated at 6 g/m^2^ wet on 35-µm Biopolymer TP302 (Film 5). These films improved the shelf life of refrigerated chicken breast meat up to seven days compared to the control sample. Moreover, all six treated films incorporated with both sage extracts (2379L/220 and 2379L/221) improved the colour stability, weight loss, texture, and pH of refrigerated chicken breast meat for up to seven days when compared to refrigerated chicken breast meat stored in the PP container. The observed antimicrobial effect is due to antimicrobial properties of sage extracts or due to overall activity of the formulated coatings. 

From this result, we found that the antimicrobial activity of the films is not only due to the different viscosity of the sage extract, but the different types of coating also play an important role. Hence, it is suggested that the coatings containing sage extracts with the different viscosity (2379L/220 and 2379L/221) can be potentially applied as antimicrobial agent for food packaging in the future.

## Figures and Tables

**Table 1:** The basic properties of the formulated coatings (adhesives) and six formulations of coated films.

The Basic Properties of Formulated Coatings (Adhesives)			
Grade	Total Solids Content (%)	Sage Solids Content (%)	Viscosity (mPa.s @ Brookfield RV, sp1, 50 rpm, 20 °C)
2379L/220	32.31	2.5	195
2379L/221	32.55	2.5	360

**Table 2:** Six formulations of coated films.

Six Formulations of Coated Films	
No	Film Code
1	Film 1—2379L/220 coated at 6 g/m^2^ wet on 12 µm PET
2	Film 2—2379L/220 coated at 6 g/m^2^ wet on 35 µm Biopolymer TP302
3	Film 3—2379L/221 coated at 6 g/m^2^ wet on 52 µm HCFD2 compostable laminate
4	Film 4—2379L/221 coated at 6 g/m^2^ wet on 12 µm PET
5	Film 5—2379L/221 coated at 6 g/m^2^ wet on 35 µm Biopolymer TP302
6	Film 6—2379L/221 coated at 6 g/m^2^ wet on 23 µm Natureflex NVR

**Table 3:** The pH value, weight loss, and hardness of chicken breast fillets stored in PP containers (control) and plastic film laminated trays during seven days of storage at 4 °C.

Property	Sample	Day			
		1	3	5	7
Weight loss (%)	Control	3.69 ± 0.03 ^Ad^	5.09 ± 0.04 ^Ac^	5.55 ± 0.04 ^Ab^	6.83 ± 0.02 ^Aa^
	Film 1	3.16 ± 0.02 ^Ed^	4.63 ± 0.02 ^Cc^	5.25 ± 0.02 ^CDb^	6.34 ± 0.02 ^Da^
	Film 2	3.23 ± 0.02 ^Dd^	4.49 ± 0.03 ^Dc^	5.11 ± 0.03 ^Eb^	6.19 ± 0.04 ^Ea^
	Film 3	3.43 ± 0.02 ^Bd^	4.59 ± 0.04 ^Cc^	5.21 ± 0.03 ^Db^	6.55 ± 0.02 ^Ca^
	Film 4	3.43 ± 0.02 ^BCd^	3.75 ± 0.02 ^Bc^	5.40 ± 0.03 ^Bb^	6.63 ± 0.02 ^Ba^
	Film 5	3.37 ± 0.03 ^Cd^	4.43 ± 0.02 ^Dc^	5.28 ± 0.04 ^CDb^	6.55 ± 0.02 ^Ca^
	Film 6	3.25 ± 0.01 ^Dd^	4.65 ± 0.02 ^Cc^	5.30 ± 0.02 ^Cb^	6.40 ± 0.02 ^Da^
Hardness (N)	Control	123.55 ± 0.01 ^Ad^	130.14 ± 0.27 ^Ac^	138.68 ± 0.14 ^Ab^	144.02 ± 0.14 ^Aa^
	Film 1	119.58 ± 0.05 ^Ed^	122.25 ± 0.08 ^Ec^	130.59 ± 0.05 ^Eb^	140.42 ± 0.11 ^Da^
	Film 2	118.80 ± 0.05 ^Fd^	121.41 ± 0.05 ^Fc^	130.22 ± 0.03 ^Fb^	137.69 ± 0.04 ^Ea^
	Film 3	120.91 ± 0.06 ^Cd^	125.79 ± 0.04 ^Cc^	133.49 ± 0.07 ^Cb^	141.50 ± 0.07 ^Ca^
	Film 4	121.38 ± 0.04 ^Bd^	125.88 ± 0.04 ^BCc^	133.97 ± 0.10 ^Bb^	142.51 ± 0.07 ^Ba^
	Film 5	121.00 ± 0.16 ^Cd^	126.19 ± 0.05 ^Bc^	134.11 ± 0.05 ^Bb^	142.43 ± 0.06 ^Ba^
	Film 6	120.23 ± 0.04 ^Dd^	122.72 ± 0.05 ^Dc^	132.43 ± 0.03 ^Db^	140.64 ± 0.15 ^Da^
pH	Control	6.22 ± 0.07 ^Ad^	6.40 ± 0.05 ^Ac^	6.81 ± 0.06 ^Ab^	7.09 ± 0.03 ^Aa^
	Film 1	6.21 ± 0.02 ^Ad^	6.28 ± 0.02 ^BCc^	6.50 ± 0.04 ^Cb^	6.75 ± 0.02 ^Ca^
	Film 2	6.22 ± 0.02 ^Ad^	6.33 ± 0.02 ^Bc^	6.45 ± 0.03 ^Cb^	6.72 ± 0.02 ^Ca^
	Film 3	6.28 ± 0.02 ^Ad^	6.34 ± 0.02 ^ABc^	6.53 ± 0.03 ^BCb^	6.83 ± 0.02 ^Ba^
	Film 4	6.24 ± 0.01 ^Ad^	6.35 ± 0.02 ^ABc^	6.63 ± 0.03 ^Bb^	6.86 ± 0.02 ^Ba^
	Film 5	6.20 ± 0.02 ^Ac^	6.26 ± 0.02 ^Cc^	6.50 ± 0.04 ^Cb^	6.76 ± 0.02 ^Ca^
	Film 6	6.25 ± 0.02 ^Ac^	6.31 ± 0.02 ^BCc^	6.52 ± 0.04 ^BCd^	6.85 ± 0.02 ^Ba^

Control: polypropylene (PP) microwavable container. Capital letters of **A**–**E** represent significant differences (*p* < 0.05) amongst samples for each day for each property. Small letters of **a**–**d** represent significant differences (*p* < 0.05) amongst storage days for each sample for each property.

## Data Availability

Not applicable.
